# Important Role of Platelets in Modulating Endotoxin-Induced Lung Inflammation in CFTR-Deficient Mice

**DOI:** 10.1371/journal.pone.0082683

**Published:** 2013-12-19

**Authors:** Caiqi Zhao, Emily M. Su, Xi Yang, Zhaowei Gao, Ling Li, Haiya Wu, Yiyi Jiang, Xiao Su

**Affiliations:** 1 Key Laboratory of Molecular Virology & Immunology, Institut Pasteur of Shanghai, Chinese Academy of Sciences, Shanghai, China; 2 Cardiovascular Research Institute, University of California San Francisco, San Francisco, California, United States of America; University of Illinois College of Medicine, United States of America

## Abstract

Mutation of CFTR (cystic fibrosis transmembrane conductance regulator) leads to cystic fibrosis (CF). Patients with CF develop abnormalities of blood platelets and recurrent lung inflammation. However, whether CFTR-mutated platelets play a role in the development of lung inflammation is elusive. Therefore, we intratracheally challenged wildtype and *F508del* (a common type of CFTR mutation) mice with LPS to observe changes of *F508del* platelets in the peripheral blood and indexes of lung inflammation (BAL neutrophils and protein levels). Furthermore, we investigated whether or not and how *F508del* platelets modulate the LPS-induced acute lung inflammation by targeting anti-platelet aggregation, depletion of neutrophils, reconstitution of bone marrow or neutrophils, blockade of P-selectin glycoprotein ligand-1 (PSGL-1), platelet activating factor (PAF), and correction of mutated CFTR trafficking. We found that LPS-challenged *F508del* mice developed severe thrombocytopenia and had higher levels of plasma TXB2 coincided with neutrophilic lung inflammation relative to wildtype control. Inhibition of *F508del* platelet aggregation or depletion of *F508del* neutrophils diminished the LPS-induced lung inflammation in the *F508del* mice. Moreover, wildtype mice reconstituted with either *F508del* bone marrow or neutrophils developed worse thrombocytopenia. Blocking PSGL-1, platelet activating factor (PAF), or rectifying trafficking of mutated CFTR in *F508del* mice diminished and alveolar neutrophil transmigration in the LPS-challenged *F508del* mice. These findings suggest that *F508del* platelets and their interaction with neutrophils are requisite for the development of LPS-induced lung inflammation and injury. As such, targeting platelets might be an emerging strategy for dampening recurrent lung inflammation in cystic fibrosis patients.

## Introduction

Recurrent inflammatory lung disease is a leading cause of morbidity and mortality in cystic fibrosis (CF), which are previously attributed to CFTR defect in the epithelial cells. However, recent studies have demonstrated that mutation of *cftr* in non-epithelial cells, for example, neutrophils and platelets, also contributes to the exaggerated proinflammatory responses of CF [1–2].

It is reported that normal human platelets and their progenitors express a biologically active CFTR [2]. Inhibition of CFTR in platelets increased phosphorylation of p38MAPK [2], and the latter propagates platelet-neutrophil interaction. In CF patients, circulating leukocyte-platelet aggregates might be related to the progression of the lung inflammation [3–5], and treatment with high-dose ibuprofen (anti-platelet aggregation) in CF patients improved lung function in the CF patients [6–7]. Platelets can interact with neutrophils through P-selectin glycoprotein ligand-1 (PSGL-1) [8] or platelet activating factor (PAF) [9] to elicit platelet aggregation, thrombocytopenia, and inflammation. However, under CFTR dysfunction, the role of platelets in modulating lung inflammation has not been validated by the animal experiments. Therefore, it is urgent to establish the relationship between platelets and lung inflammatory profiles in the *F508del* CFTR mice when they are exposed to inflammatory insults.

We have reported *F508del* neutrophils play a key role in mediating LPS (endotoxin, E. coli lipopolysaccharide)-induced acute lung inflammation and injury [2]. In that study, we found that *F508del* neutrophils were more proinflammatory than the wildtype neutrophils. Wildtype mice reconstituted with *F508del* bone marrow or neutrophils developed a neutrophilic lung inflammation and edema when the mice were intratracheally challenged with LPS [2]. In line with those findings, we raised the following questions: (i) Were there any platelet abnormalities during LPS-induced lung inflammation in *F508del* mice? If the platelets were highly activated, whether anti-platelet aggregation could alter the pulmonary inflammatory responses in the LPS-challenged *F508del* mice? (ii) Can wildtype mice recapitulate the phonotype of *F508del* mice, such as, platelet abnormalities and lung inflammation, by adoptively transferring the *F508del* bone marrow or neutrophils to the wildtype mice? (iii) Would blockade of PSGL-1 or PAF affect thrombocytopenia and lung inflammation in *F508del* mice? (iv) Can correction of the trafficking of *F508del* CFTR reverse platelet abnormalities and lung inflammation of *F508del* mice?

Therefore, the overall objective of this study is to test whether *F508del* platelets play a role in the development of LPS-induced lung inflammation by targeting the following strategies: anti-platelet aggregation, depletion of neutrophils, reconstitution of bone marrow or neutrophils, blockade of PSGL-1 or PAF, and correction of mutated CFTR trafficking. The findings will provide us a novel way of dampening lung inflammation in the CF patients.

## Materials and Methods

### Reagents

LPS (*E. coli* 0111:B4) and Aspirin (dissolved in DMSO) were from Sigma-Aldrich (St. Louis, MO). PE-conjugated PSGL-1 antibody or PE-conjugated isotype control antibody were purchased from BD Pharmingen (San Diego, CA). Anti-Gr-1 (clone RB6-8C5), anti-PSGL-1, and corresponding isotype (G2b, κ) antibodies were from the UCSF Cell Facility. WEB 2086 (a platelet activating factor receptor antagonist) and KM 11060 (a corrector of *F508del* CFTR trafficking) were from Tocris Bioscience (Ellisville, MO).

### Animals

Eight to ten-week old CD1 wild-type and CF mice (targeted *F508del* gene replacement, obtained from Professor A. Verkman, University of California San Francisco) were used for these studies [1]. The CF mice were back-crossed into a CD1 genetic background (>8 generations) and bred at the University of California, San Francisco animal facility. CF mice were genotyped in accordance with standard procedures [10]. Anesthesia was induced with an intraperitoneal injection (IP) of a mixture of ketamine (90 mg/kg) and xylazine (10 mg/kg). The Committee on Animal Research of the University of California, San Francisco and Institut Pasteur of Shanghai, Chinese Academy of Sciences approved all the protocols.

### LPS-induced acute lung inflammation mouse model

A previously developed direct visualization instillation (DVI) method [11] was used to instill LPS into the airspaces of the lung. The LPS dosage (5 mg/kg) was chosen aiming to induce a robust lung inflammation and injury at 24 h as previously reported [1] and no mice died at this dosage.

### Bronchoalveolar lavage (BAL) neutrophil and protein measurements

BAL was done after euthanizing the mice and then placing a 20-gauge catheter into the trachea through which 1 ml of cold PBS was flushed back and forth three times. BAL neutrophils were also analyzed by a multispecies hematology instrument (Hemavet 950FS; Drew Scientific, Dallas, TX). BAL cytology was prepared by cytospin staining (Cytospin 3; Thermo Electron). Protein concentration was measured in the BAL fluid from all experimental groups, as an index of lung endothelial and epithelial permeability (Bio-Rad protein assay kit, Hercules, CA).

### Measurement of platelets in blood

A sample of blood was placed in EDTA-coated vials (BD Microtainer) and Hemavet analyzer was used to generate blood platelet counts.

### Neutrophil isolation

*F508del* and CD1 wildtype mice were euthanized and the bone marrow from the femurs and tibias was flushed with PBS using a 25-gauge needle. The whole bone marrow was centrifuged and washed in PBS, and the erythrocytes were hypotonically lysed with 0.2% NaCl. This solution was restored to isotonicity with 1.2% NaCl and then filtered over a 70-µm nylon cell strainer (BD Biosciences-Discovery Labware). The solution was centrifuged and resuspended in PBS and then applied over a 62% Percoll gradient. The Percoll solution was centrifuged for 30 min at 1,500×g. The neutrophil pellet was then isolated, washed, and centrifuged twice, and counted with a Coulter counter. Greater than 90% neutrophil purity was confirmed with a cytospin preparation and Hema 3 staining.

### Neutrophil depletion and reconstitution

Neutrophil depletion was accomplished with a rat anti-mouse Gr-1 monoclonal antibody (Gr-1 mAb) which was purified from the RB6-8C5 hybridoma. The Gr-1 mAb (160 µg) was given intraperitoneally (IP), yielding greater than 90% neutrophil depletion at 24 h. Before neutrophil-depleted mice were intratracheally challenged with LPS (5 mg/kg), 6×10^6^ freshly isolated bone marrow neutrophils were intravenously injected to reconstitute blood neutrophils as previously described [12].

### Bone marrow chimera experiments

Mice were lethally irradiated (6 Gy) and immediately reconstituted via retro-orbital injection with either wildtype or *F508del* bone marrow (5×10^6^ cells). Mice were administered antibiotic-treated water for 3 weeks and then used for experiments at approximately 8 weeks after bone marrow transfer. Using this protocol, at 8 weeks after bone marrow transplantation, more than 90% of circulating hematopoietic cells are of donor origin.

### Measurements of plasma TXB2 by ELISA

Plasma TXB2 levels were separately assayed using by TXB2 Enzyme Immunoassay Kit (Assay Design, Ann Arbor, MI).

### Flow cytometry

Blood mononuclear cells were isolated by *Ficoll-Paque™* (GE Healthcare, Piscataway, NJ) density centrifuge. BAL cells (pooled from five BAL samples in each group) were incubated directly with PE-conjugated PSGL-1 antibody or PE-conjugated isotype control antibody. The labeled cells were analyzed by flow cytometry, with gating on the basis of forward and side scatter. Flow cytometric acquisition was performed using a FACScan (BD Biosciences; Cytek Development Inc.), and data were analyzed using Summit Software (Dako Colorado Inc.).

### Statistical analysis

Statistics were done by the SPSS software (SPSS Inc., Chicago, IL) and results are presented as means ± SD. One-way analysis of variance (ANOVA) with post hoc Bonferroni test or Student's t test were used (level set at P<0.05).

## Results

### Severe thrombocytopenia in F508del mice coincided with neutrophilic lung inflammation

**Figure 1:**
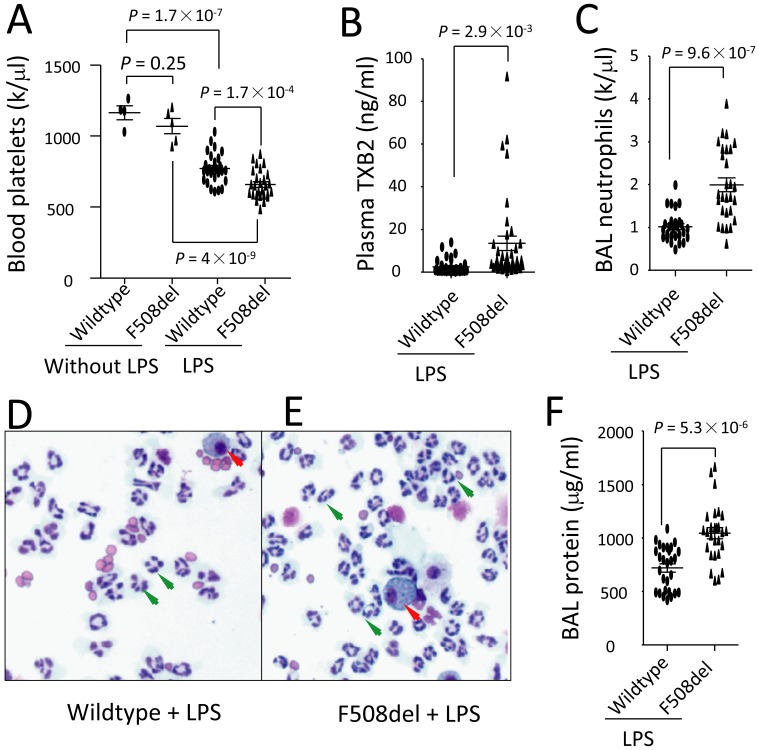
Reduction of blood platelets coincides with worsened lung inflammation in F508del mice that were intratracheally challenged with LPS. Wild-type and CFTR mutated mice were intratracheally challenged with either PBS (for Figure 1A) or LPS. The mice were killed at 24 h after challenge. A. Blood platelet counts were accessed (n = 4–5 in before LPS group; n = 27, in LPS-challenged group); B. Plasma TXB2 levels were measured by ELISA (n = 37, in each group); C–E. BAL neutrophils (n = 28, in each group) counted by Hemavet analyzer and cytological examination (Wright's stain; magnification ×100; red arrow head indicates alveolar macrophage; green arrow head indicates neutrophils); F. BAL protein levels (an indicator of lung endothelial and epithelial permeability, n = 28, in each group). Data were pooled from more than 5 independent experiments with LPS challenge. Data were analyzed by ANOVA and t-test and presented in mean ± SD. P values are listed on each panel.

There is no significant difference in blood platelet counts between wildtype and *F508del* mice before LPS challenge (Figure 1 A). BAL neutrophils and protein levels in the naïve wildtype and *F508del* mice did not differ as published previously [1]. Wildtype and *F508del* mice were intratracheally challenged with LPS. At 24 h, platelet counts in the blood dropped in both wildtype and *Fdel508* mice, but there was a more significant reduction in *F508del* mice compared to wildtype mice (Figure 1 A). Plasma TXB2 levels markedly increased in *F508del* mice under LPS challenge (Figure 1 B). These findings demonstrate that *F508del* platelets are quantitatively changed in respond to LPS challenge. Concurrently, lung inflammation was severe in *F508del* mice relative to the wildtype reflected by more neutrophils present in the BAL (Figure 1 C–E) and higher BAL protein levels (an index of lung epithelial and endothelial permeability, Figure 1 F). These findings suggest that changes of *F508del* platelets might contribute to neutrophilic lung inflammation induced by LPS.

**Figure 2:**
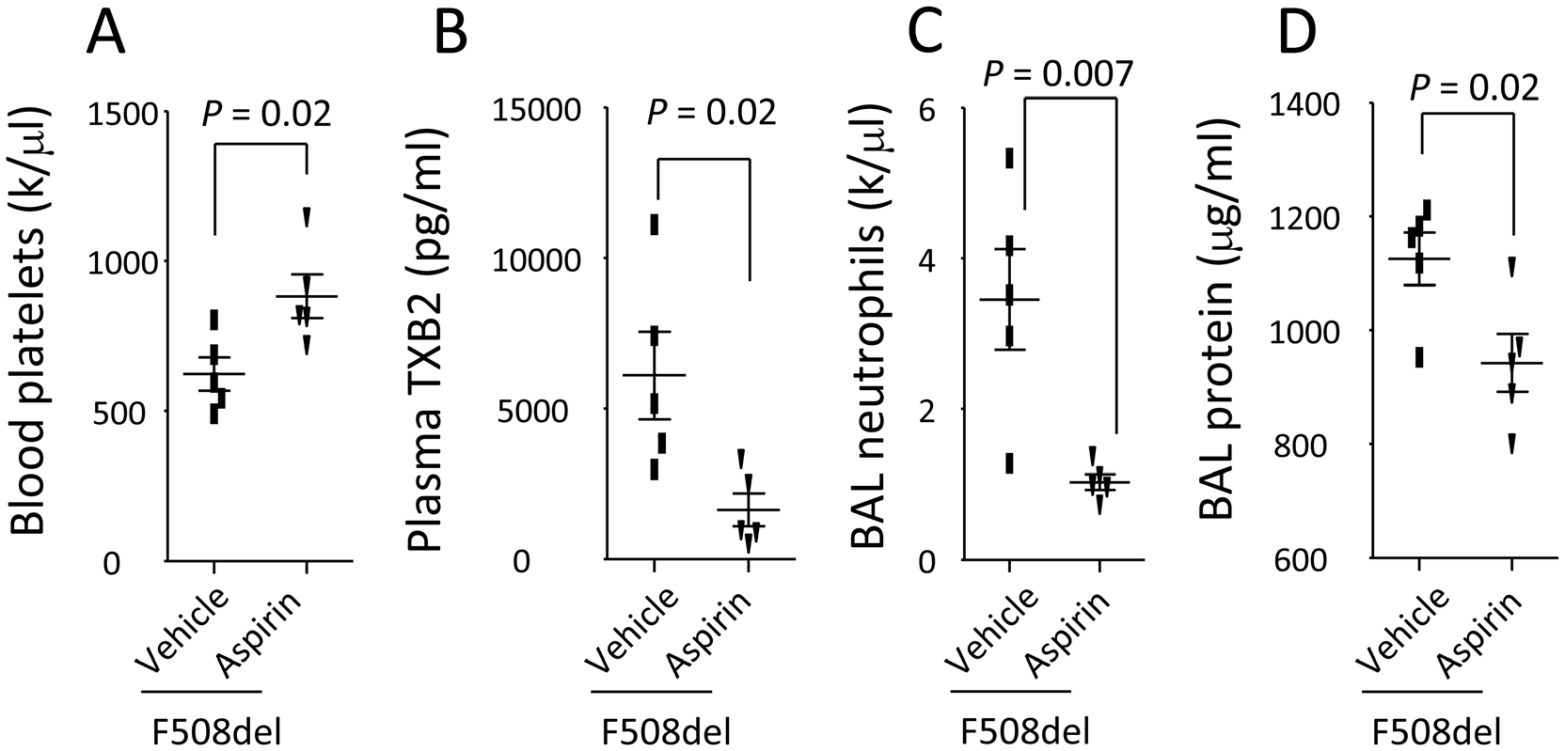
Prevention of platelet aggregation by Aspirin reverses thrombocytopenia and reduces LPS-induced lung inflammation in F508del mice. A. Pretreatment with Aspirin increased blood platelet counts; B. Pretreatment with Aspirin reduced plasma TXB2 levels in LPS-challenged *F508del* mice compared to LPS-challenged *F508del* mice receiving vehicle; C–D. Administration of aspirin reduced BAL neutrophils and protein levels in LPS-challenged *F508del* mice compared to LPS-challenged *F508del* mice receiving vehicle. N = 5 in each group. Data were analyzed by t-test and presented in mean ± SD. P values are listed on each panel.

It should be noted that most of the experiments in the current study were performed in four groups: wildtype + pretreatment with vehicle, wildtype + pretreatment with agent, *F508del* + pretreatment with vehicle, and *F508del* + pretreatment with agent. We have found that wildtype and *F508del* mice received pretreatment(s) with vehicle(s) and then challenged with LPS displayed the same phenotype as the data showed in the Figure 1. To present briefly, in the Figures 2, 4–7, we only show the data obtained from the LPS-challenged *F508del* mice that received either vehicle(s) or the corresponding pretreatment(s) with interventions.

### Inhibition of platelet aggregation reduces LPS-induced lung inflammation in F508del mice

We hypothesize that platelet aggregation manifested by thrombocytopenia would contribute to the development of LPS-induced lung inflammation. To test this hypothesis, we pretreated the *F508del* mice intraperitoneally with either vehicle or aspirin (a classical anti-platelet aggregation agent), and then challenged them with LPS. At 24 h, thrombocytopenia was inverted by anti-platelet therapy, and this notion is supported by a significant increase of peripheral platelet counts (Figure 2 A) and a marked reduction of plasma TXB2 (a metabolite of TXA2, inducer of platelet aggregation) in the aspirin-pretreated group (Figure 2 B). Anti-platelet aggregation by aspirin significantly reduced LPS-induced lung inflammation, reflected by a marked reduction of BAL neutrophils and protein levels in the aspirin-pretreated group (Figure 2 C–D). These findings strongly support that thrombocytopenia mediated by *F508del* platelet aggregation facilitates the development of LPS-induced lung inflammation.

### F508del thrombocytopenia and TXB2 production are dependent on bone marrow-derived cells, specifically neutrophils

**Figure 3:**
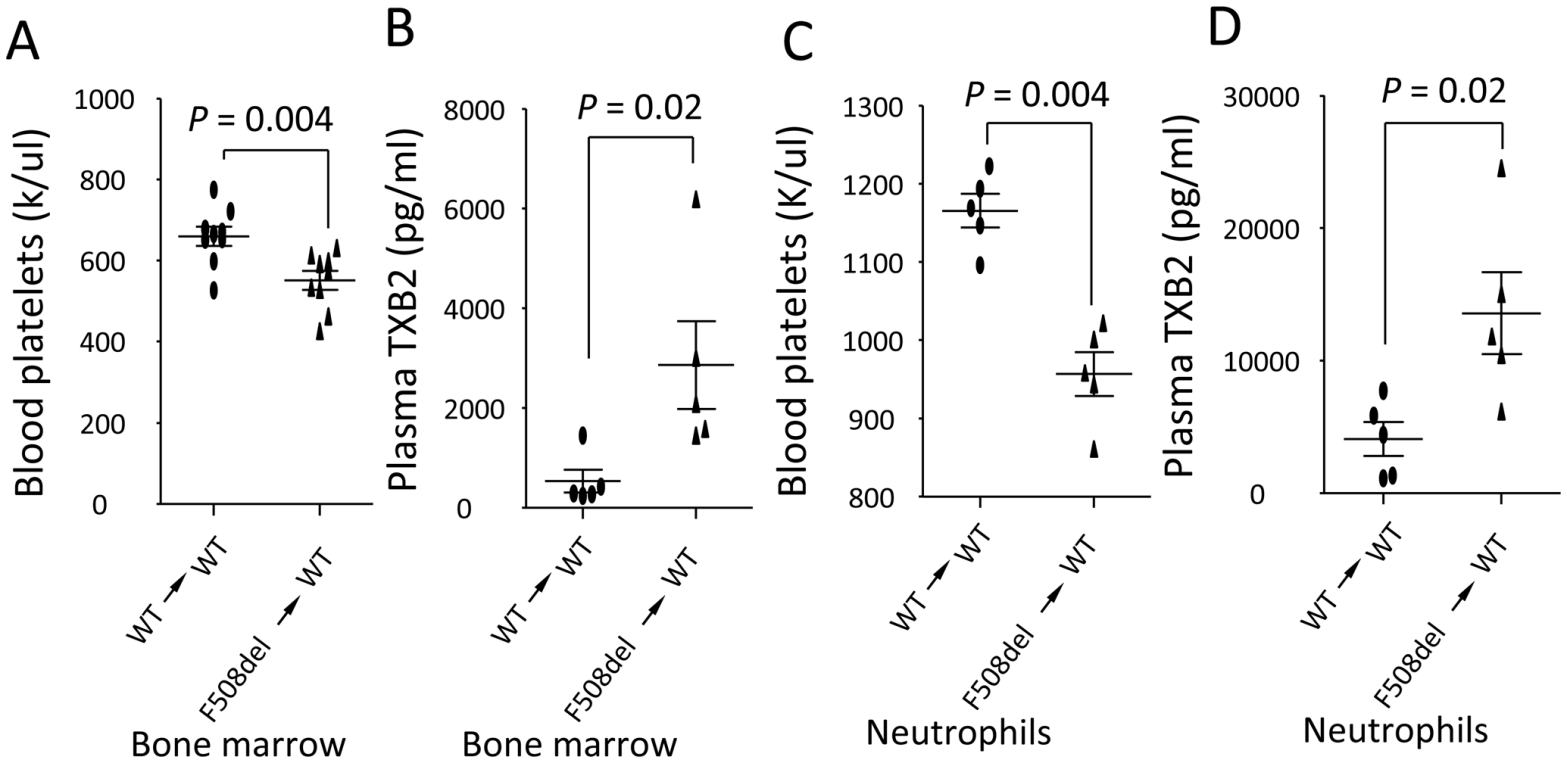
F508del bone marrow-derived cells, specifically neutrophils, play an important role in mediating thrombocytopenia. A–B. Wildtype mice were irradiated and then reconstituted with wildtype or *F508del* bone marrow to create chimeric mice. At 24 h after LPS challenge, wildtype mice receiving *F508del* bone marrow developed thrombocytopenia (A) (N = 9 in each group) with higher plasma TXB2 levels (B) (N = 5 in each group) compared to wildtype mice receiving wildtype marrow. C–D. Wildtype mice were given anti-Gr-1 antibody to deplete neutrophils and then reconstituted with either wildtype or *F508del* neutrophils. 24 h after LPS challenge, wildtype mice receiving *F508del* neutrophils developed thrombocytopenia (C) and had higher levels of plasma TXB2 (D). N = 5 in each group. Data were analyzed by t-test and presented in mean ± SD. P values are listed on each panel.

We hypothesized that bone marrow-derived cells (eg. neutrophils, platelets) are important for mediating LPS-induced lung inflammation. To test this hypothesis, we irradiated the wildtype mice and then reconstituted them with wildtype or *F508del* isolated bone marrow cells. Three months later, these chimeras were challenged with LPS. At 24 h, in response to LPS challenge wildtype mice repopulated with *F508del* bone marrow cells developed thrombocytopenia (Figure 3 A) with higher plasma TXB2 levels (Figure 3 B) compared to wildtype mice repopulated with wildtype bone marrow cells, suggesting that *F508del* bone marrow-derived cells might account for thrombocytopenia and production of TXB2 in the peripheral blood during LPS-induced lung inflammation. To specify that *F508del* neutrophils are required to induce thrombocytopenia and TXB2 production, wildtype mice were given Gr-1 antibody to depletion neutrophils and then reconstituted (iv) with either wildtype or *F508del* neutrophils. Under LPS challenge, blood platelets reduced (Figure 3 C) and plasma TXB2 levels (Figure 3 D) increased in wildtype mice receiving *F508del* neutrophils compared to wildtype mice receiving wildtype neutrophils, suggesting that *F508del* neutrophils might contribute to production of TXB2 and thrombocytopenia.

### Depletion of F508del neutrophils reduces thrombocytopenia and lung inflammation

**Figure 4:**
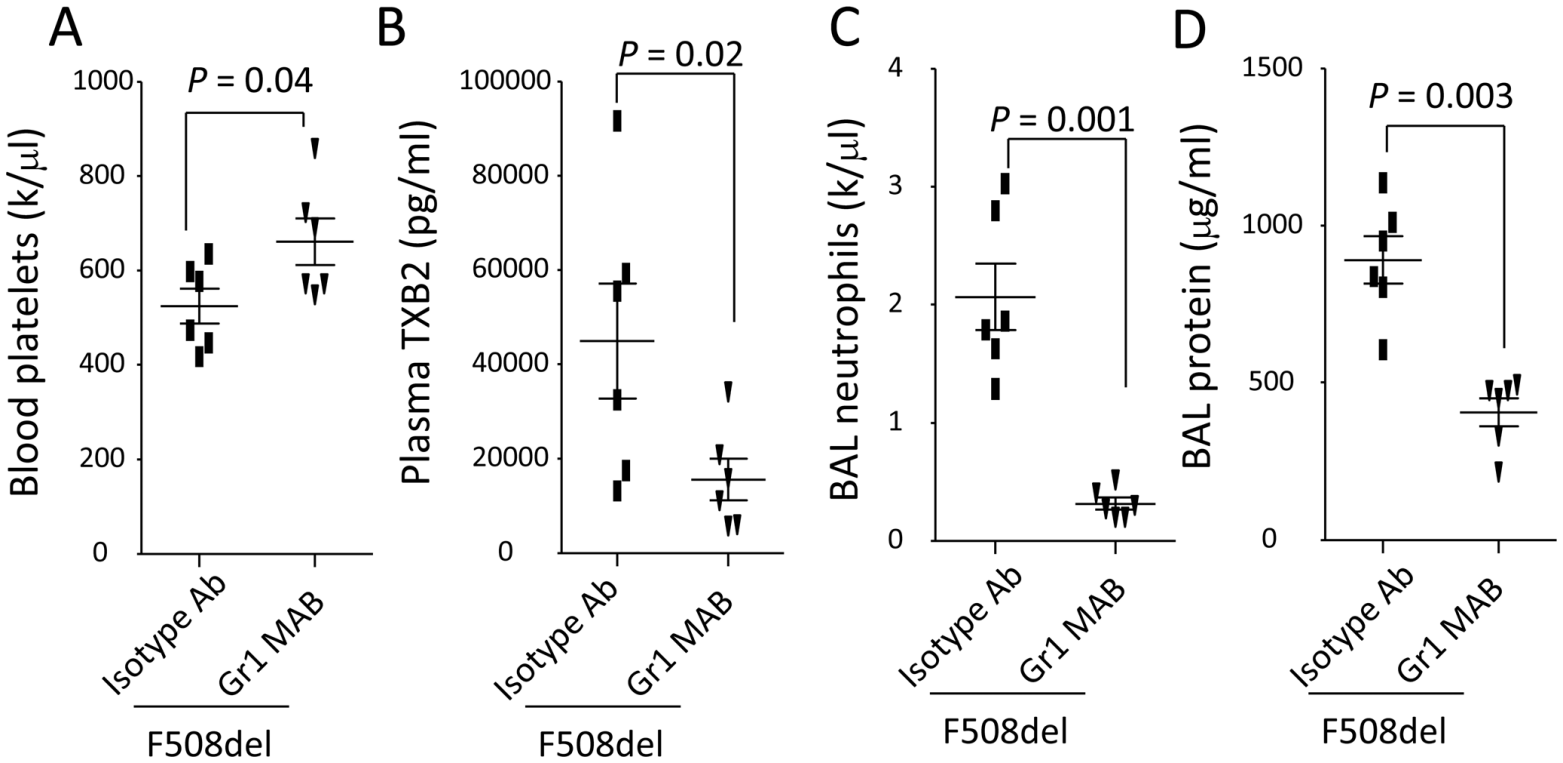
Depletion of neutrophils reverses thrombocytopenia and LPS-induced lung inflammation in F508del mice. **A–B**. Under intratracheal LPS challenge, depletion of neutrophils in *F508del* mice reversed thrombocytopenia (**A**) and reduced plasma TXB2 levels (***B***) compared to without neutrophil depletion. **C–D**. Depletion of neutrophils *in F508del* mice decreased BAL neutrophils (**C**) and protein levels (**D**) compared to without neutrophil depletion. N = 6 in each group. Data were analyzed by t-test and presented in mean ± SD. P values are listed on each panel.

To demonstrate *F508del* neutrophils are sufficient to induce thrombocytopenia and lung inflammation, *F508del* mice were intravenously given anti-Gr-1 or isotype antibodies. Then, these two groups of mice were intratracheally challenged with LPS. At 24 h, pretreatment with anti-Gr-1 antibody in *F508del* mice reversed thrombocytopenia (Figure 4 A) and reduced plasma TXB2 levels (Figure 4 B) compared to *F508del* mice receiving isotype antibody. Both BAL neutrophils (Figure 4 C) and protein levels (Figure 4 D) were reduced in *F508del* mice receiving anti-Gr-1 antibody compared to *F508del* mice receiving isotype antibody, suggesting that *F508del* neutrophils are sufficient to promote thrombocytopenia and lung inflammation.

### F508del neutrophil migration and thrombocytopenia depend on PSGL-1

**Figure 5:**
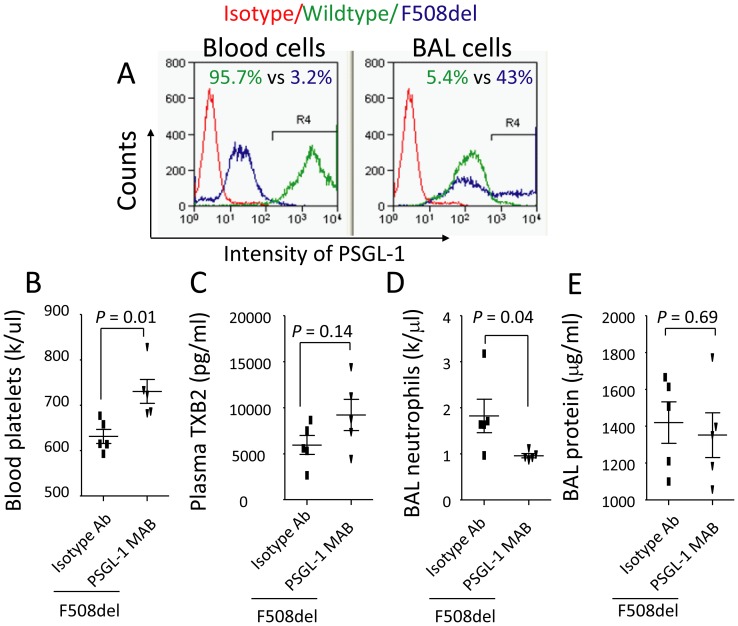
Blockade of PSGL-1 prevents thrombocytopenia and neutrophil transalveolar migration in F508del mice. **A**. Analysis of PSGL-1^+^ leukocytes in blood and BAL cells by flow cytometry. Cells were isolated from wild-type and *F508del* mice after LPS challenge at 24 h and then stained with PE anti-PSGL-1 antibody. **B–C**. Blockade of PSGL-1 reversed thrombocytopenia induced by LPS in *F508del* mice (**B**), but did not affect plasma TXB2 levels (**C**). **D**. Blockade of PSGL-1 reduced BAL neutrophils in F508del mice, but did not affect BAL protein levels (**E**). N = 5 in each group. Data were analyzed by t-test and presented in mean ± SD. P values are listed on each panel.

To compare transmigration of *F508del* PSGL-1^−^ and PSGL-1^+^ leukocytes from the peripheral blood to the alveoli, wildtype and *F508del* mice were intratracheally instilled with LPS, PSGL-1^+^ cells were numerated in both blood and BAL cells by PE-PSGL-1 antibody labeling. At 24 h, fewer PSGL-1^+^ cells presented in the blood and more PSGL-1^+^ cells migrated into the alveoli in *F508del* mice compared to wild-type (Figure 5 A), suggesting that *F508del* PSGL-1^+^ leukocytes more efficiently migrate from peripheral blood to the alveoli.

To corroborate that interaction between neutrophils and platelets through PSGL-1 mediates thrombocytopenia and neutrophil migration, *F508del* mice were first treated intravenously with either anti-PSGL-1 or isotype antibody, then intratracheally instilled with LPS. At 24 h, administration of anti-PSGL-1 antibody prevented reduction of blood platelets (Figure 5 B), but did not affect the plasma TXB2 levels (Figure 5 C), suggesting anti-PSGL-1 antibody directly disrupts interaction of between neutrophils and platelets and prevents thrombocytopenia. Consequently, the BAL neutrophils were reduced in the PSGL-1 antibody-treated *F508del* mice compared to isotype antibody treated group (Figure 5 D), reinforcing the result that PSGL-1 play an important role in mediating *F508del* neutrophil alveolar transmigration. However, blockade of PSGL-1 did not influence the lung vascular permeability revealed by the BAL protein levels did not differ in these two groups (Figure 5 E).

### Blockade of PAF receptor diminishes thrombocytopenia and lung inflammation in F508del mice

**Figure 6:**
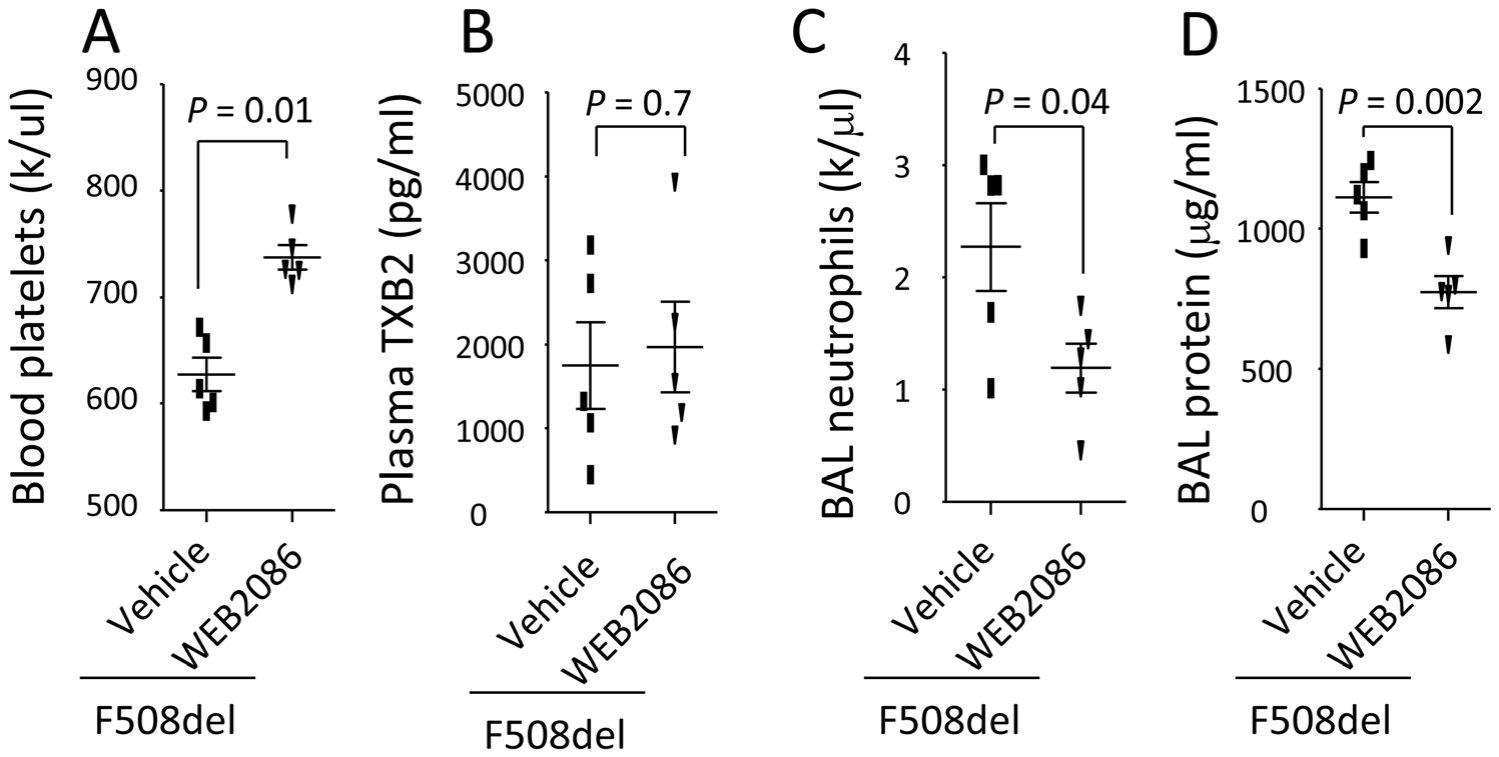
Blockade of PAF by WEB2086 prevents thrombocytopenia in F508del and lung inflammation induced by LPS. **A**. Blockade of PAF by WEB2086 *prevented thrombocytopenia*; but did not affect plasma TXB2 levels (**B**). **C–D**. Blockade of PAF by WEB2086 reduced BAL neutrophils (**C**) and protein levels (**D**) in *F508del* mice under LPS challenge. N = 5 in each group. Data were analyzed by t-test and presented in mean ± SD. P values are listed on each panel.

To demonstrate PAF is required for mediating thrombocytopenia and neutrophil alveolar transmigration, *F508del* mice were first treated (iv) with either WEB2086 (a PAF receptor antagonist) or vehicle, then intratracheally instilled with LPS. At 24 h, WEB2086 increased blood platelets (Figure 6 A), suggesting PAF is required for development of thrombocytopenia in *F508del* mice. But, WEB2086 did not affect the plasma TXB2 levels (Figure 6 B). Consequently, the BAL neutrophils (Figure 6 C) and protein levels (Figure 6 D) were reduced in WEB2086-treated *F508del* mice compared to vehicle treated group, indicating that PAF promotes LPS-induced thrombocytopenia and lung inflammation in *F508del* mice.

### Correction of F508del CFTR reduces thrombocytopenia, neutrophil transmigration, and lung inflammation

**Figure 7:**
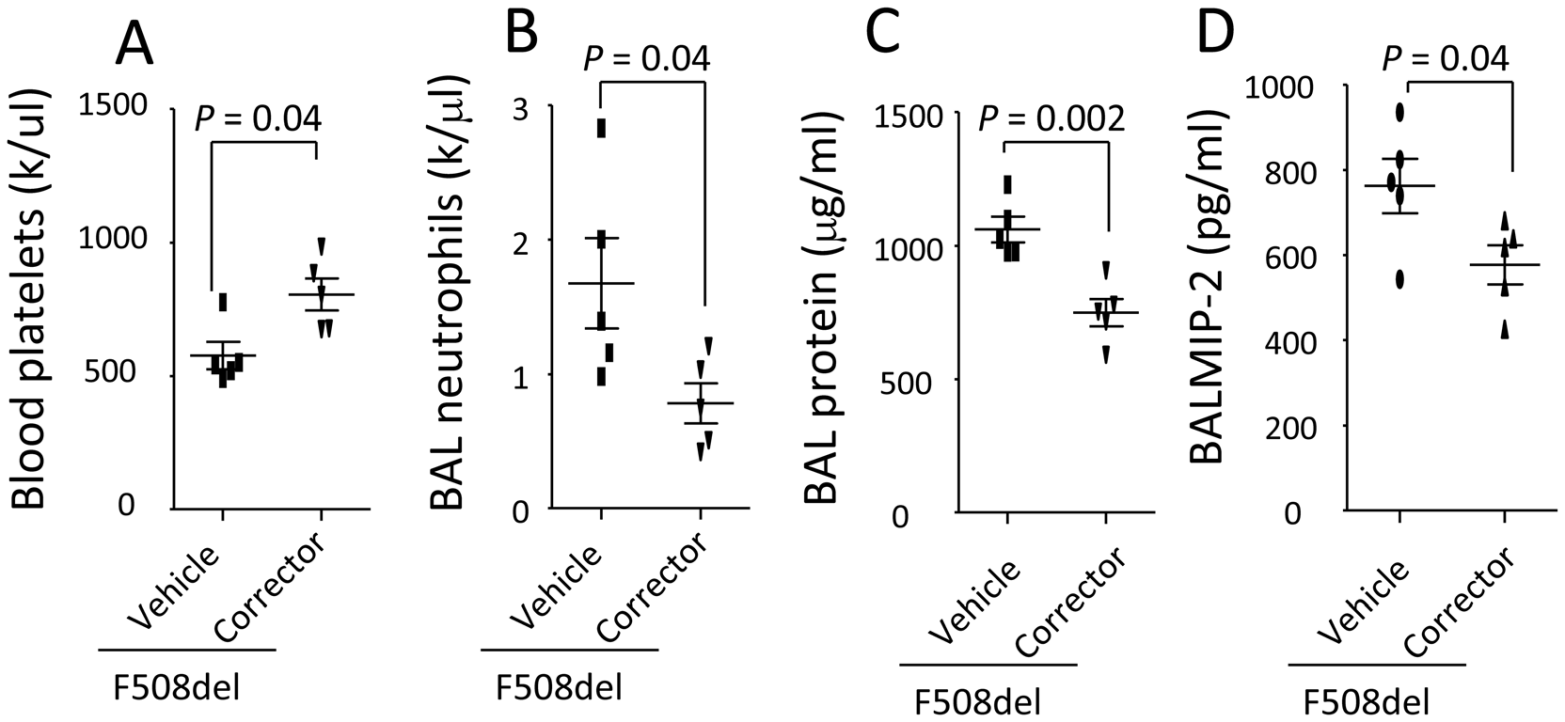
Correction of F508del CFTR trafficking by KM 11060 prevents thrombocytopenia in F508del and lung inflammation induced by LPS. A. Correction of *F508del* CFTR trafficking by KM11060 *prevented thrombocytopenia.* B–D. Rectification of *F508del* CFTR trafficking by KM11060 reduced BAL neutrophils (B), protein levels (C), and MIP-2 levels (D) in *F508del* mice under LPS challenge. N = 5 in each group. Data were analyzed by t-test and presented in mean ± SD. P values are listed on each panel.

To test whether correction of mutated *F508del* CFTR can prevent thrombocytopenia and neutrophil migration, two groups of *F508del* mice were separately pretreated with KM 11060 or vehicle and then intratracheally challenged with LPS. In LPS-challenged *F508del* mice, KM 11060 lessened thrombocytopenia (Figure 7 A), decreased BAL neutrophils (Figure 7 B) and protein levels (Figure 7 C), and BAL MIP-2 levels (Figure 7 D) compared to the vehicle-treated group in response to intratracheal LPS challenge.

## Discussion

In this study, we have found that in *F508del* mice developed thrombocytopenia and worse lung inflammation compared to wildtype mice in response to intratracheal LPS challenge. Attenuation of thrombocytopenia by inhibiting platelet aggregation, depleting *F508del* neutrophils, blocking PSGL-1 or PAF, or rectifying of *F508del* CFTR trafficking in LPS-challenged *F508del* mice significantly diminished neutrophil transalveolar migration or lung inflammation. These findings suggest *F508del* platelets play an important role in modulating LPS-induced acute lung inflammation.

The most important finding of this study is that in response to LPS challenge, *F508del* mice developed thrombocytopenia and severe lung inflammation manifested by higher BAL neutrophils and protein levels compared to wildtype mice. Our result is different from the previous clinical research that CF patients have an increase in circulating activated platelets determined by leukocyte-platelet aggregation [4]. In that study, (i) 32 CF patients were recruited, but the complications of these patients were unclear; (ii) Platelets in the peripheral blood were not numerated. Platelet activation was examined in 18 out of 32 recruited patients by flow cytometry; (iii) FEV1%, a parameter of lung function, was listed; however, the data largely varied from different patients; and (iv) Relationship between blood platelet activation and lung inflammation and function was not clearly established. In this study, for the first time, we demonstrate that thrombocytopenia corresponds to worse lung inflammation in the *F508del* mice when exposing to LPS compared to wildtype mice.

The second important finding of our study is that anti-platelet aggregation by Aspirin through inhibiting cyclooxygenase (COX) pathway can markedly diminish LPS-induced thrombocytopenia and lung inflammation in the *F508del* mice (Figure 2). This finding supports that thrombocytopenia is strongly associated with development of lung inflammation, which provides experimental evidence to support the prior findings that administration of ibuprofen can improve the lung function in the CF patients [6–7]. This finding further directs us to test whether and how interplay between platelets and neutrophils determine the outcome of CF lung inflammation.

Previous studies have showed that neutrophils might contribute to recurrent lung inflammation in the CF patients [13–14]. We also demonstrated that neutrophils are requisite for deterioration of LPS-induced lung inflammation in the *F508del* mice relative to wildtype [1]. Based on these findings, we hypothesize that *F508del* neutrophils might collaborate with platelets to define the LPS-induced acute lung inflammation, and this notion is supported by the findings that (i) thrombocytopenia and lung inflammation was markedly reduced after *F508del* neutrophils were depleted; (ii) wildtype mice reconstituted with *F508del* bone marrow or neutrophils developed more severe thrombocytopenia and lung inflammation when exposed to the LPS (Figures 3–4). Although Gr-1 antibody (RB6-8C5) also deplete some monocytes [12], the reconstitution with *F508del* neutrophils or repopulation of bone marrow-derived cells in the wildtype mice could recapitulate LPS-induced inflammatory features as the *F508del* mice owned. These results strongly support that *F508del* neutrophils promote platelet activation and aggregation, thrombocytopenia, which mediate LPS-induced lung inflammation.

To further study that interaction between *F508del* neutrophils and platelets is required for development of thrombocytopenia, neutrophil alveolar transmigration, and lung inflammation, we assessed PSGL-1 and PAF, which are two key molecules that contribute to neutrophil-platelet aggregation and neutrophil transmigration. Evidence has shown that PSGL-1 expressed by neutrophils is a dominant ligand for P-selectin present in platelets and endothelial cells [15]. We have found that (i) PSGL-1 expressed by *F508del* leukocytes was engaged during transmigration of PSGL-1^+^ leukocytes [16–17] (Figure 5 A) in LPS-induced lung inflammation; (ii) blockade of PSGL-1 prevented thrombocytopenia (Figure 5 B) and reduced transmigration of neutrophils to the alveoli (Figure 5 D). These findings collectively support that *F508del* leukocytes and platelets collaborate to induce platelet aggregation and neutrophil transmigration during LPS-induced lung inflammation.

Studies have shown that PAF synthesis is increased in response to bacterial endotoxin both *in vivo* and *in vitro* 18–19. PAF itself or combining with LPS can promote platelet activation and recruitment of neutrophils in the endotoxin-induced lung injury [20–21]. In this study, we demonstrate that blockade of PAF by WEB 2086 reversed thrombocytopenia and limited neutrophil alveolar transmigration, which also reinforces that *F508del* neutrophils interacting with platelets through PAF determines the outcome of LPS-induced acute lung inflammation (Figures 6 A, C–D).

Previous study showed that platelets were a source of TXB2 [22]. We found that thrombocytopenia paralleled with higher plasma levels of TXB2 in LPS-challenged *F508del* mice (Figure 1 A–B). Administration of Aspirin can inhibit COX-1 and reduce production of TXB2 (Figure 2 B). Especially, wildtype mice reconstituting with *F508del* neutrophils or bone marrow had higher levels of plasma TXB2 compared to wildtype mice receiving wildtype neutrophils or bone marrow (Figure 3 B, 3 D), conversely, depleting *F508del* neutrophils reduced plasma TXB2 in LPS-challenged *F508del* mice (Figure 4 B), suggesting that *F508del* neutrophils are the main generators of TXB2. Therefore, *F508del* neutrophils through generating COX-1 product contribute to LPS-induced lung inflammation and injury [1]. However, anti-PSGL-1 or blockade of PAF by WEB 2086 did not affect plasma TXB2 levels, suggesting PSGL-1 or PAF signaling-induced *F508del* platelet aggregation is not related to COX-1 pathway.

As our previously study shown [1], defect of CFTR in neutrophils worsens LPS-induced lung inflammation and injury. In our unpublished data, alveolar macrophages also express CFTR. Inhibition or deletion of CFTR in the alveolar macrophages facilitates proinflammatory cytokine production induced by LPS. Therefore, we cannot rule out that Aspirin and other reagents used in the current study only act on platelets thereby mediating LPS-induced lung inflammation and injury.

In summary, *F508del* platelets exert an unexpectedly modulating role in the development of LPS-induced lung inflammation. More importantly, our data have supported that correction of the *F508del* CFTR trafficking [23] are able to counteract the proinflammatory phenotype of *F508del* platelets and neutrophils, reflected by attenuation of thrombocytopenia, reduction of BAL neutrophils, protein, and MIP-2 levels. As such, targeting platelets might be a promising strategy for dampening persistent lung inflammation in cystic fibrosis patients.
